# Thermal Kinetics of Monocationic and Dicationic Pyrrolidinium-Based Ionic Liquids

**DOI:** 10.3390/ma15031247

**Published:** 2022-02-08

**Authors:** Asyraf Hanim Ab Rahim, Noraini Abd Ghani, Noorhafizah Hasanudin, Normawati M. Yunus, Ninna Sakina Azman

**Affiliations:** 1 Centre of Research in Ionic Liquids, Universiti Teknologi PETRONAS, Seri Iskandar 32610, Perak, Malaysia; asyrafhanim92@gmail.com (A.H.A.R.); fiezah_hfzh@yahoo.com (N.H.); normaw@utp.edu.my (N.M.Y.); ninna.azman@gmail.com (N.S.A.); 2 Fundamental and Applied Sciences Department, Universiti Teknologi PETRONAS, Seri Iskandar 32610, Perak, Malaysia

**Keywords:** ionic liquids, monocationic, dicationic, thermal kinetic, activation energy

## Abstract

This work presents an in-depth kinetic thermal degradation comparison between traditional monocationic and the newly developed dicationic ionic liquid (IL), both coupled with a bromide (Br^−^) anion by using non-isothermal thermogravimetric analysis. Thermal analyses of 1-butyl-1-methylpyrrolidinium bromide [C_4_MPyr][Br] and 1,4-bis(1-methylpyrrolidinium-1-yl)butane dibromide [BisC_4_MPyr][Br_2_] were conducted at a temperature range of 50–650 °C and subjected to various heating rates, which are 5, 10, 15, 20 and 25 °C/min. Thermogravimetric analysis revealed that dicationic IL, [BisC_4_MPyr][Br_2_] is less thermally stable compared to monocationic [C_4_MPyr][Br]. A detailed analysis of kinetic parameters, which are the activation energy (*E_a_*) and pre-exponential factor (*log A*), was calculated by using Kissinger-Akahira-Sunose (KAS), Flynn-Wall-Ozawa (FWO) and Starink. This study revealed that the average *E_a_* and *log A* of [BisC_4_MPyr][Br_2_] are lower than [C_4_MPyr][Br], which may be contributed to by its low thermal stability. Conclusively, it proved that the *E_a_* and *log A* of ILs are strongly related to the thermal stability of ILs.

## 1. Introduction

The first ever ionic liquids (ILs) were founded by Paul Walden in 1914, as he discovered ethyl ammonium-nitrate [EtNH_3_][NO_3_], which had a melting point less than 12 °C [1]. This discovery has become a foundation that inspires researchers to synthesize various ILs, such as 1-ethylpyridinium bromide-aluminium chloride [C_2_Py][Br-AlCl_3_], 1-butylpyridinium chloride-aluminium chloride [C_4_Py][Cl-AlCl_3_] and 1-buty-3-methylimidazolium chloride [2,3,4]. Basically, ILs are a molten salt consisting of cations and anions and have a melting point lower than 100 °C [5]. In the late 20th century, works related to ILs demonstrated increasing trends, mainly due to its unique properties, such as high thermal stability, wider liquidous range and negligible vapor pressure [6]. Apart from that, the properties of Ils, namely, thermal stability, viscosity, hydrophobicity and conductivity, can be changed according to the application of interest, due to the availability of various cation and anion combinations [7].

The thermal stability of ILs is an important parameter that should be considered before ILs are applied to specific application. Apart from the academic perspective, the thermal stability of ILs is crucial in some applications involving high temperature, such as transesterification and dissolution. According to Xu and Cheng, the thermal stability studies of ILs are usually conducted by using UV-Vis spectroscopy, flame ionization detection (FID) and mass spectrometry (MS) [8]. Nevertheless, the most popular technique involves the determination of onset temperature (*T_o_*), which is measured by using a thermogravimetric analyzer (TGA) [9,10]. Through TGA, an isoconversional kinetic study can be conducted due to its reliability of providing a detailed evaluation for the determination of kinetic parameters [11]. There are three methods based on the iso-conversional kinetic: maximum rate, differential and integral isoconversional. A review by Xu and Cheng stated that although differential isoconversional methods based on Friedman provide *E_a_* values corresponding to each α, the *E_a_* calculation indicates a poor fitting in results and linearity [8]. In addition, the *E_a_* calculated by the Friedman method also demonstrates a broader variation range. Meanwhile, the integral isoconversional method, utilized by Starink and Coats-Redfern (CR) and consisting of the Flynn-Wall-Ozawa (FWO) and Kissinger-Akahira-Sunose (KAS) methods, is more accurate than the differential method [8].

The structure of anions and cations provides a major role in the thermal stability of ILs [12,13]. Nowadays, the search for thermally stable ILs is constantly being carried out—until recently, dicationic ILs emerged as a new category. Several papers reported that dicationic ILs have superior properties in terms of their thermal stability compared to traditional monocationic ILs [14,15]. This due to their higher liquid density, intermolecular interaction and molecular weight [8,16], thus allowing the utilization of dicationic ILs as a lubricant, gas chromatography stationary phase, catalyst for trans-esterification pro-cess and solar cell [17,18]. Theoretically, in terms of an intermolecular interaction, there is a larger number of hydrogen bonds in dicationic ILs than monocationic Ils, due to the presence of more hydrogen atoms [19]. This bond is very stable, thus contributing to high thermal stability of dicationic ILs. Until now, numerous studies reported the thermal pyrolysis of kinetic study for mono- and dicationic imidazolium-based ILs [12,20,21]. However, the thermal kinetic data for ILs with non-aromatic rings, especially pyrrolidinium-based ILs, is still scarce, although these types of ILs have been applied in numerous applications. For example, Quraishi et al. reported the use of the pyrollidinium-based IL with a different alkyl chain to absorb CO_2_ to be used in microalgae photosynthesis [22]. Furthermore, Lombardo and co-workers applied *N*-butyl *N*-methyl pyrrolidinium triflate ([bmpyr][TfO]) as a catalyst for a direct asymmetric aldol reaction [23].

**Figure 1:**
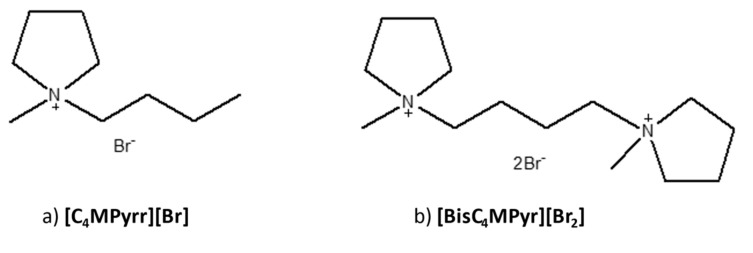
The chemical structure of (**a**) [C_4_MPyr][Br] and (**b**) [BisC_4_MPyr][Br_2_].

In light of this, two pyrolidium-based ILs, which are 1-butyl-1-methylpyrrolidinium bromide [C_4_MPyr][Br] and 1,4-bis(1-methylpyrrolidinium-1-yl)butane dibromide [BisC_4_MPyr][Br_2_], as shown in Figure 1, was synthesized according to the method suggested by Burrel et al. and Montalbán et al. [24,25], with a slight modification in terms of the reactants ratio. Both ILs were characterized using various characterization techniques, including NMR, CHNS and TGA. Additionally, the integral isoconversional method was selected to determine the kinetic parameters of ILs, which are the activation energy (*E_a_)* and pre-exponential factors (*log A)* at different heating rates. The effect of mono and dicationic structure of ILs towards IL’s thermal stability was also discussed.

## 2. Materials and Methods

### 2.1. Materials

All solvents and chemical reagents were purchased from Merck, Darmstadt, Germany and used without any purification. The CAS number, source and grade of the chemicals are as follows: 1-bromobutane (109-65-9, Merck, 99%), 1-methylpyrrolidine (120-94-5, Merck, 98%), 1,4-dibromobutane (110-51-1, Merck, 99%), 2-propanol (67-63-0, Merck, 99.5%) and diethyl ether (60-29-7, Merck, 99.9%).

### 2.2. Instrumentations

#### 2.2.1. Structural Characterization

^1^H and ^13^C characterizations of ILs were recorded on a Bruker Advance III (500MHz) Nuclear Magnetic Resonance (NMR) spectrometer (Bruker, Billerica, MA, USA) using deuterated solvent, D_2_O. About 80 µL of the IL sample was added into a NMR tube containing 550 µL D_2_O. The measurement was conducted at room temperature and the chemical shift was reported in parts per million (ppm) with TMS as an internal standard. The multiplicities were abbreviated as s = singlet, d = duplet, t = triplet and m = multiplet.

#### 2.2.2. Elemental Analysis

The percentage of carbon (C), hydrogen (H) and nitrogen (N) in ILs were analyzed by using CHNS Elementar Vario Micro Cube with infrared analyzer (Elementar Analysensyteme GmbH, Langenselbold, Germany). About 2.0 mg of the IL sample was weighed by a highly precise balance in aluminium foil and sealed in a silver capsule. The sample was loaded in an autosampler to be analyzed. Before the analysis, the instrument was calibrated by using sulfonamide. The measurement was conducted at 1000 °C and the reduction furnace temperature was 650 °C. Helium was used as the carrier gas.

#### 2.2.3. Thermal Pyrolysis of ILs

The thermogravimetric analysis (TG) of the ILs was measured using STA 6000 from Perkin Elmer, Waltham, MA, USA. In this work, about 5.0 mg of sample was weighed in a crucible pan and placed on the sample holder. The measurement was conducted at five different heating rates, which are 5, 10, 15, 20 and 25 °C/min, to study the degree of pyrolysis in the temperature range of 50–650 °C under 20 mL/min nitrogen flow. The condition for thermal pyrolysis was selected based on a preliminary analysis on the IL’s degradation, also based on the method suggested by Masri et al. and Meng et al. [26,27]. In this study, the thermal analysis for kinetic pyrolysis was done twice under the same conditions to verify the reliability of the results.

### 2.3. Experimental

#### 2.3.1. Synthesis of Monocationic IL; 1-butyl-1-methylpyrrolidinium bromide [C_4_MPyr][Br]

The synthesis of monocationic [C_4_MPyr][Br] was conducted based on the method suggested by Burrel and co-workers [24]. An equal molar of 1-bromobutane (8.05g, 0.059 mole) was slowly added into a round bottom flask containing methylpyrrolidine (5 g, 0.059 mole) that was stirred at 150 rpm. The reaction was conducted under a solventless condition. The mixture was refluxed at 40 **°**C for 24 h. A total of 15 mL of diethyl ether was added into the resulting product and the mixture was moderately shaken to remove unreacted reactants. The step was repeated for three times before the resulted clear yellowish liquid, then was dried in a vacuum oven for 24 h (89.9%). **^1^H NMR** (500 MHz, D_2_O) δ **=** 1.81 (m, 4H, CH_2_), 2.13 (s, 8H, CH_2_-N), 2.97 (s, 6H, CH_3_), 3.33 (t, 4H, CH_2_-N), 3.45 (m, 8H, CH_2_). Theoretical calculation (%): C, 48.66; H, 9.07; N, 6.30, Experimental: C, 48.24; H, 9.13; N, 6.45.

#### 2.3.2. Synthesis of Dicationic IL; 1,4-bis(1-methylpyrrolidinium-1-yl)butane dibromide [BisC_4_MPyr][Br_2_]

The synthesis of dicationic [BisC_4_MPyr][Br_2_] was conducted based on the method provided by Montalbán et al. [25]. An equal molar of 1,4-dibromobutane (10.8 g, 0.05 mole) was slowly added into a round bottom flask containing a mixture of methylpyrrolidine (4.3 g, 0.05 mole) and 2-propanol and stirred at 150 rpm. The mixture was refluxed at a temperature of 70 °C for 24 h. Then, 2-propanol was evaporated in vacuo, resulting in highly viscous dark brown IL. The resulting IL was washed with 15 mL diethyl ether for three times before being dried under a vacuum to yield a solid product (86.0%). ^1^H NMR (500 MHz, D_2_O) δ = 1.97 (t, 4H), 2.24 (s, 8H), 3.084 (s, 6H), 3.08 (s, 6H), 3.40 (t, 4H), 3.58 (m, 8H). Theoretical calculation (%): C, 43.54; H, 7.83; N, 7.25. Experimental: C, 43.62; H, 8.0; N, 7.65.

### 2.4. Kinetic Thermal Decomposition

The kinetics of the mono and di-IL decomposition reaction was determined by using the Starink, KAS and FWO methods. These three methods were selected as they are frequent p(y)-isoconversion methods, which allows the measurement of kinetic parameters, *E_a_* and *log A* for at different heating rates. Each method produced a thermokinetic based on equations by KAS, FWO and Starink, as depicted in Equations (1)–(3), respectively [26].

(1) $$ln\left(\frac{\beta }{T^{2}}\right)=ln\left(\frac{AR}{E_{a}g\left(\alpha \right)}\right)-\frac{E_{a}}{RT}\;$$

(2) $$ln\beta =ln\left(\frac{AE_{a}}{Rg\left(\alpha \right)}\right)-5.331-\frac{1.052E_{a}}{RT}\;$$

(3) $$ln\left(\frac{\beta }{T^{1.92}}\right)=ln\left(\frac{AR^{0.92}}{g\left(\alpha \right)E_{a}^{0.92}}\right)-0.312-\frac{1.008E_{a}}{RT}\;\;$$

where *β* represents heating rates, *T* is the absolute temperature, *g*(*α*) is a function conversion factor, *A* is the pre-exponential factor, *R* is the ideal gas constant of 8.314 J/mol, while *E_a_* is the activation energy. The calculation to obtain *E_a_* and *log A* is performed by using the average temperature obtained in each α value, as provided in the supplementary data.

## 3. Results and Discussions

### 3.1. Thermal Decomposition Analysis

**Figure 2:**
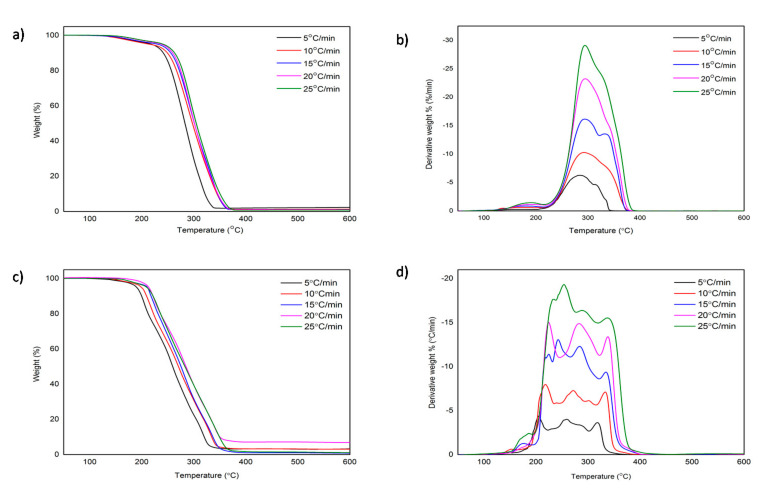
The TG and DTG curves for (**a**,**b**) [C_4_MPyr][Br] and (**c**,**d**) [BisC_4_MPyr][Br_2_] at heating rate of 5–25 **°**C/min.

In this work, the thermal stability of [C_4_MPyr][Br] and [BisC_4_MPyr][Br_2_] was reported in term of onset temperature (*T_o_*) and decomposition temperature (*T_max_*). *T_o_* is an intersection that exists between the baseline and the tangent of sample weight vs. temperature, whereas *T_max_* is the temperature in which the maximum weight loss of the sample was recorded [10]. The thermogravimetric (TG) curves of mono- [C_4_MPyr][Br] and dicationic [BisC_4_MPyr][Br_2_] ILs were compared in Figure 2a,c. Meanwhile, the *T_o_* for both ILs at heating rates of 5 to 25 °C/min are shown in Table 1. Based on Table 1, it is demonstrated that the *T_o_* for [C_4_MPyr][Br] is higher than [BisC_4_MPyr][Br_2_]. Generally, this indicates a better thermal stability of mono-[C_4_MPyr][Br] compared to [BisC_4_MPyr][Br_2_]. This result is opposed to studies performed by Bender et al. and Fareghi et al., in which they reported a better thermal stability for dicationic imidazolium-based ILs [28,29].

The difference in the thermal behavior of [BisC_4_MPyr][Br_2_] compared to other dicationic ILs could be caused by its chemical structure, which consists of heterocyclic non-aromatic pyrrolidinium instead of aromatic imidazolium and pyridinium. Theoretically, the improvement of thermal stability for dicationic ILs with aromatic heterocyclic structures is due to the delocalization and the presence of π bonds in its entire ring system. Therefore, the presence of the double aromatic in dicationic ILs will significantly improve their thermal stability. However, for ILs in this study, the presence of two weak non-aromatic heterocylic pyrrolidinium rings may contribute to the low thermal stability of [BisC_4_MPyr][Br_2_]. Other than that, the presence of the more powerful nucleophile Br^-^ in [BisC_4_MPyr][Br_2_] compared to [C_4_MPyr][Br] may also cause the decrease in the thermal stability of [BisC_4_MPyr][Br_2_]. At 25 °C, the density of mono-[C_4_MPyr][Br] is 1.1997 cm^−3^, which is larger than dicat-[BisC_4_MPyr][Br_2_], with a density value of 1.4726 cm^−3^. This result was in line with work conducted by Shirota and co-workers, in which ILs with high densities possessed better thermal stability compared with ILs with low densities [16].

However, unlike [C_4_MPyr][Br], with a single peak at its derivative thermogravimetric (DTG) curve, the DTG curve of [BisC_4_MPyr][Br_2_] displays the presence of several peaks, which suggests that multiple stages have occurred in the decomposition process. However, only the highest peak in DTG was selected for further analysis [30]. It is revealed that the *T_max_* of both ILs also reflects the same result as *T_o_*, where the *T_max_* for [C_4_MPyr][Br] and [BisC_4_MPyr][Br_2_] are in the range of 283–294 °C and 205–254 °C, respectively. In addition, as shown in Table 1, an increase in heating rates increased the *T_o_* and *T_max_* of Ils, which is in agreement with the data reported in the previous literature [20]. This is due to the increase of heat supply into the system, as when the heating rate increased, faster chemical reaction kinetics were caused [31].

**Figure 3:**
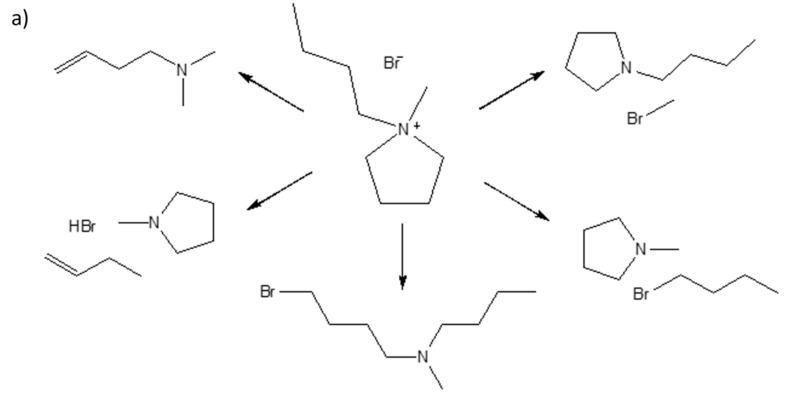

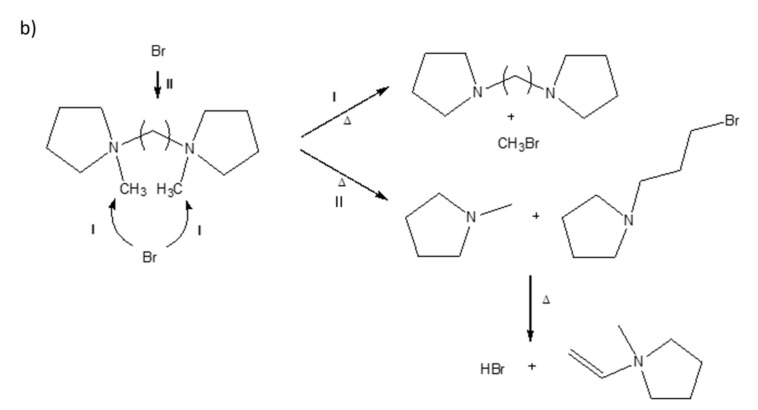
The postulated thermal degradation mechanism of (**a**) [C_4_MPyr][Br] and (**b**) [BisC_4_MPyr][Br_2_].

In the meantime, although there is a difference between the DTG pattern of [C_4_MPyr][Br] and [BisC_4_MPyr][Br_2_], a similar pattern of behavior of the TG and DTG curves was observed for each individual IL, regardless of the different heating rates. This is attributed to the similar decomposition mechanism possessed by each ILs. Cao and Mu stated that the degradation of ILs consists of decomposition and evaporation processes [10]. In the decompostion stage, the formation of new substances occur due to the nucleophilic substitution reaction, SN_2_, and is followed by the transformation of the sample into a gaseous state [32]. In the meantime, Patil et al. and Wooster et al. provided a detailed study on the mechanisms for ILs decomposition by analyzing pyrolysis products using mass spectrometry [33,34]. Generally, the thermal degradation mechanism of ILs mainly involved reverse Menshutkin reactions and Hofmann eliminations [34]. The attack of the nucleophilic Br^-^ anion towards the cation moiety led to the loss of alkyl substituents on the pyrolle ring. Meanwhile, at the side chain, Hofmann elimination may contribute to the formation of terminal alkenes and protonated anions [34]. Figure 3a,b demonstrates the postulated degradation mechanism of [C_4_MPyr][Br] and [BisC_4_MPyr][Br_2_].

### 3.2. Kinetic of Thermal Decomposition

**Figure 4:**
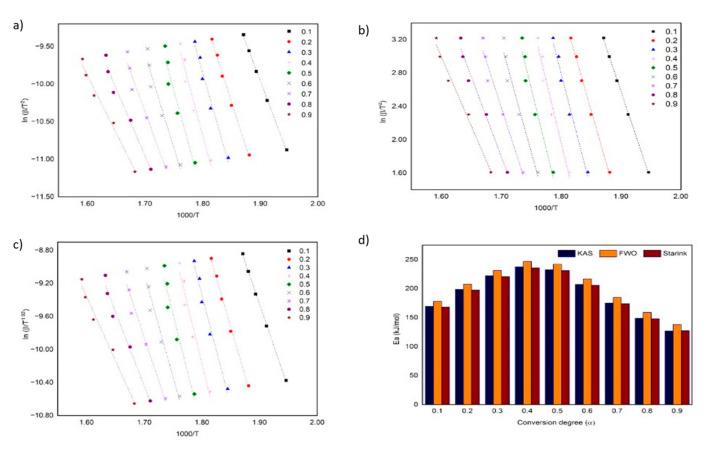
Thermokinetic plot using (**a**) KAS, (**b**) FWO, (**c**) Starink method and (**d**) the *E_a_* profile for [C_4_MPyr][Br].

Theoretically, the kinetic of the thermal decomposition parameters for ILs can be calculated by using the TG and DTG approach. In this study, the kinetic parameters, namely, *E_a_* and *log A*, were calculated by using the TG approach, and it was determined based on a fraction conversion (α) by applying the KAS, FWO and Starink methods. Table 2 shows the correlation coefficient values (*R*^2^) of the three models exceeding 0.9, which indicates a high degree of linearity [31]. Figure 4 shows the thermokinetic plot for KAS, FWO and Starink, including *E_a_*, for the monocationic IL, [C_4_MPyr][Br], at different α. The average *E_a_* of [C_4_MPyr][Br], as deduced by KAS, FWO and Starink, are 191 ± 38, 201 ± 38 and 190 ± 38 kJ/mol, respectively. Moreover, the pre-exponential factor was presented in terms of the *log A*, in which the values for KAS, FWO and Starink are 17 ± 4, 18 ± 4 and 17 ± 4 min^−1^. According to Guida and co-workers, the slight difference in *E_a_* and *log A* values obtained is due to an improper approximation of temperature integration [35]. Based on Figure 4d, the three models show similar *E_a_* trends from α values of 0.1–0.9. An increasing trend of *E_a_* was observed from α values of 0.1–0.4 before it is reduced to a range of 0.5–0.9. The increase in *E_a_* is due to the IL partial decomposition, which normally occurs in the beginning of the thermal process at low temperatures. Meanwhile, a reduction in *E_a_* indicates the formation of intermediates due to the SN_2_ nucleophilic substitution reaction [26].

**Figure 5:**
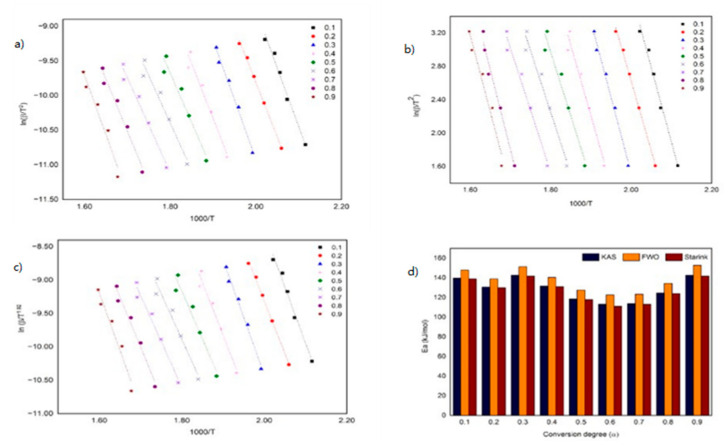
Thermokinetic plot using (**a**) KAS, (**b**) FWO, (**c**) Starink method and (**d**) the *Ea* profile for [BisC_4_MPyr][Br_2_].

In the meantime, Figure 5 shows the kinetic plot and *E_a_* profile for [BisC_4_MPyr][Br_2_]. The average *E_a_* for KAS, FWO and Starink are 129 *±* 12, 138 *±* 12 and 128 *±* 12 kJ/mol, respectively. Based on Figure 4d, the *E_a_* trend of [BisC_4_MPyr][Br_2_] fluctuates as the α value increases. A work by Masri and co-workers also revealed the same trend for their ([DABCODBS][HSO_4_]_2_) and ([DABCODBS][CF_3_SO_3_]_2_. It was suggested that the fluctuating trend of *E_a_* happened due to the multi-step thermal decomposition process [26]. The average *log A* values of [BisC_4_MPyr][Br_2_] are 12 *±* 2, 14 *±* 2 and 12. *±* 1 min^−1^. Further analysis of the kinetic parameters of both ILs demonstrated that [BisC_4_MPyr][Br_2_] owns lower *E_a_* and log A values compared to [C_4_MPyr][Br]. This could be related to the low thermal stability of [BisC_4_MPyr][Br] compared to [C_4_MPyr][Br]. Furthermore, the low *E_a_* of [BisC_4_MPyr][Br_2_] suggested that it had a faster chemical reaction than [C_4_MPyr][Br]. While the Starink and KAS method produced almost identical *E_a_* and *log A* values for both ILs, the *E_a_* obtained by using FWO is slightly higher. The *E_a_*, *log A* and *R^2^* values for both ILs were presented in Table 2. On the other hand, Bender and co-workers had performed the kinetic decomposition study on mono- and dicationic imidazolium-based ILs coupled with the Br^-^ anion by using the FWO method [28]. It was determined that the values of kinetic parameters for their imidazolium-based dicationic ILs at α *=* 0.1 and α *=* 0.5 are higher than our dicationic pyrollidinium ILs. This indicates that the energy needed by dicationic imidazolium-based ILs for their reactants to reach the transition state is higher than the dicationic pyrollidinium-based ILs.

Data on the maximum operation temperature (MOT) for pyrrolidinium-based ILs are still scarce. Several recent studies used MOT to predict the long-term thermal stability successfully in several recent studies [36,37,38]. Furthermore, the thermal stability of the ILs mixture is predicted using MOT [39]. The values of the kinetic parameters obtained from this work can be utilized to predict the MOT of ILs. The MOT of [C_4_MPyr][Br] and [BisC_4_MPyr][Br_2_] was calculated based on Equation (4), as follows [40]:

(4) $$MOT=\frac{E_{a}/R}{4.6+\ln\left(A.t_{max}\right)}$$

where *t_max_* is the maximum time of exposition. Table 3 shows the *MOT* of [C_4_MPyr][Br] and [BisC_4_MPyr][Br_2_] that was calculated by using kinetic parameters obtained from KAS, FWO and Starink.

## 4. Conclusions

This work aims to investigate the thermal stability, *E_a_* and *log A*, for monocationic and dicationic pyrrolidinium-based ILs. The experimental data on thermal stability indicates low thermal stability of dicationic [BisC_4_MPyr][Br_2_] compared to the monocationic [C_4_MPyr][Br]. The DTG analysis had also revealed the single and multistage decomposition of [C_4_MPyr][Br] and [BisC_4_MPyr][Br_2_], respectively. Furthermore, the kinetic evaluation work was conducted using the KAS, FWO and Starink method, in which the value of *R^2^* obtained is more than 0.9, thus proving that each α is best fitted with all the selected kinetic equations. Based on the TG data analysis, it can be concluded that the IL thermal stability does provide a great influence on the *E_a_* and *log A* values. It was determined that the *E_a_* and *log A* are linearly proportional with thermal stability of the ILs. In the meantime, while the average values of *E_a_* for [C_4_MPyr][Br], obtained by using KAS, FWO and Starink, are 191 ± 38, 201 **±** 38 and 190 ± 38 kJ/mol, the average [BisC_4_MPyr][Br_2_] *E_a_* are 129 ± 12, 138 ± 12 and 128 ± 12 kJ/mol. The same case was also observed in terms of the *log A* value for both ILs. However, the *E_a_* and *log A* value provided by the FWO model was found to be 10% higher than KAS and Starink, thus suggesting it was quite unreliable. The utilization of the monocationic [C_4_MPyr][Br] and dicationic [BisC_4_MPyr][Br_2_] as catalysts in transesterification has not yet been explored. Therefore, this study is crucial in order to provide preliminary information related to the thermal stability and kinetic property of pyrollidium-based ILs as a catalyst.

## Figures and Tables

**Table 1:** T_o_ and T_max_ at different heating rates.

Heating Rate/°C min^−1^	[C_4_MPyr][Br]		[BisC_4_MPyr][Br_2_]	
	*T_o_* (°C)	*T_max_* (°C)	*T_o_* (°C)	*T_max_* (°C)
5	255 ± 10	297 ± 20	191 ± 1	208 ± 3
10	253 ± 2	292 ± 1	201 ± 4	218 ± 2
15	259 ± 1	293. ± 1	206 ± 3	237 ± 7
20	265 ± 2	290 ± 5	206 ± 3	236 ± 14
25	265 ± 3	293 ± 1	213 ± 4	251 ± 5

**Table 2:** Values of E_a_, log A and *R^2^* for [C_4_MPyr][Br] and BisC_4_MPyr][Br_2_] at different fraction conversions.

Method	*α*	*E_a_* (kJ/mol)		*log A* (min^−1^)		*R* ^2^	
		[C_4_MPyr][Br]	[BisC_4_MPyr][Br_2_]	[C_4_MPyr][Br]	[BisC_4_MPyr][Br_2_]	[C_4_MPyr][Br]	[BisC_4_MPyr][Br_2_]
KAS	0.1	169.28	139.79	15.79	14.03	0.9991	0.9837
	0.2	198.85	130.61	18.45	12.88	0.9953	0.9969
	0.3	222.03	142.82	20.51	13.89	0.9778	0.9885
	0.4	237.51	131.72	21.76	12.39	0.9544	0.9594
	0.5	232.59	118.52	21.03	10.80	0.941	0.9464
	0.6	207.17	113.32	18.41	10.06	0.9475	0.9585
	0.7	175.08	113.77	15.22	9.86	0.9601	0.9715
	0.8	148.97	124.55	12.63	10.58	0.9739	0.9739
	0.9	127.96	142.71	10.55	11.90	0.9846	0.956
FWO	0.1	177.99	147.83	15.77	14.10	0.9991	0.9894
	0.2	207.83	138.88	18.33	13.04	0.9957	0.9973
	0.3	231.19	151.35	20.30	14.00	0.9794	0.9898
	0.4	246.81	140.53	21.51	12.60	0.9576	0.9642
	0.5	242.04	127.59	20.81	11.12	0.9452	0.9535
	0.6	216.76	122.63	18.30	10.43	0.9518	0.9645
	0.7	184.85	123.34	15.27	10.25	0.9640	0.9757
	0.8	158.92	134.41	12.83	10.92	0.9770	0.9776
	0.9	138.12	152.87	10.90	12.16	0.9868	0.9616
Starink	0.1	168.28	139.00	16.83	15.07	0.9991	0.9837
	0.2	197.62	129.91	19.19	13.63	0.9953	0.9969
	0.3	220.63	142.02	21.06	14.46	0.9779	0.9885
	0.4	236.00	131.02	22.18	12.84	0.9545	0.9596
	0.5	231.13	117.93	21.37	11.15	0.9412	0.9467
	0.6	205.90	111.14	18.66	12.65	0.9477	0.9588
	0.7	174.08	113.25	15.42	10.07	0.9603	0.9717
	0.8	148.18	123.96	12.78	10.73	0.9741	0.9741
	0.9	127.35	141.97	10.65	15.07	0.9847	0.9563

**Table 3:** The MOT for [C_4_MPyr][Br] and BisC_4_MPyr][Br_2_].

Methods	[C_4_MPyr][Br]/°C	[BisC_4_MPyr][Br_2_]/°C
KAS	409.73	398.29
FWO	400.73	375.67
Starink	435.42	423.68

## Data Availability

The data that support the findings in the present study are available from the corresponding author upon request.

## Supplementary Materials

The following supporting information can be downloaded at: https://www.mdpi.com/article/10.3390/ma15031247/s1, Table S1: Average temperature of [C_4_MPyr][Br] at each α value of thermal kinetic decomposition; Table S2: Average temperature of [BisC_4_MPyr][Br] at each α value thermal kinetic decomposition.
