# Supplementary material for: Thermal Kinetics of Monocationic and Dicationic Pyrrolidinium-Based Ionic Liquids
Source: Materials (Basel). 2022 Feb 8;15(3):1247. doi: 10.3390/ma15031247 (PMC8840748; doi:10.3390/ma15031247)
Supplement: Supplementary file 1 [file materials-15-01247-s001.zip › materials-1525605-supplementary.pdf]

materials

MDPI

Supplementary Materials

Thermal Kinetics of Monocationic and Dicationic Pyrrolidinium-Based Ionic Liquids

Asyraf Hanim Ab Rahim [1], Noraini Abd Ghani [1,2,*], Noorhafizah Hasanudin [1], Normawati M. Yunus [1,2] and Ninna Sakina Azman [1,2]

1 Centre of Research in Ionic Liquids, Universiti Teknologi PETRONAS, Seri Iskandar 32610, Perak, Malaysia; asyrafhanim92@gmail.com (A.H.A.R.); fiezah_hfzh@yahoo.com (N.H.); normaw@utp.edu.my (N.M.Y.); ninna.azman@gmail.com (N.S.A.)
2 Fundamental and Applied Sciences Department, Universiti Teknologi PETRONAS, Seri Iskandar 32610, Perak, Malaysia
* Correspondence: noraini.ghani@utp.edu.my

Table S1. Average temperature of [C_4MPyr][Br] at each α value of thermal kinetic decomposition.

	Rate (°C/min)				
α	5	10	15	20	25
0.1	241±1	250±0.7	255±2	259±1	261±3
0.2	258±2	268±0.7	272±0.5	275±0.5	277±2
0.3	269±3	278±0.3	282±0.4	284±1	286±2
0.4	278±4	287±0.5	292±0.05	292±1	295±2
0.5	286±5	296±2	301±0.3	302±0.5	303±2
0.6	295±5	305±4	312±0.8	312±3	313±1
0.7	3030±4	314±6	323±0.8	324±5	326±1
0.8	311±2.10	324±10	335±1	338±9	339±4
0.9	321±0.4	334±14	347±3	353±9	355±7

Table S2. Average temperature of [BisC_4MPyr][Br] at each α value thermal kinetic decomposition.

	Rate (°C/min)				
α	5	10	15	20	25
0.1	199±0.5	209±0.07	213±7	216±8	222 ±0.2
0.2	212±0.04	222±0.6	228±5	232±6	237±0.6
0.3	229±2	237±2	243±5	249±7	251±0.5
0.4	244±3	254±2	260±2	269±2	268±2
0.5	258±3	269±2	274±2	287±3	285±3
0.6	270±3	283±3	289±2	302±4	301±4
0.7	285±3	298±4	306±4	318±4	318±3
0.8	303±2	315±6	323±4	334±2	336±4
0.9	323±4	331±5	340±5	350±0.23	353±5
